# Specific genotypes of human papillomavirus in 125 high-grade squamous lesions and invasive cervical cancer cases from Congolese women

**DOI:** 10.1186/1471-2458-14-1320

**Published:** 2014-12-23

**Authors:** Luc Magloire Anicet Boumba, Lahoucine Hilali, Mustapha Mouallif, Donatien Moukassa, Moulay Mustapha Ennaji

**Affiliations:** Laboratoire de Virologie, Microbiologie et Qualité/ETB, Faculté des Sciences et Techniques, Université Hassan II Mohammedia, B.P. 146, Mohammedia, 20650 Maroc; Laboratoire d’Agroalimentaire et Santé, Département de Biologie Appliquée, Faculté des Sciences et Techniques, Université Hassan 1er Settat, B.P. 577, Settat, Maroc; Laboratoire d’Analyses Médicales et Morphologiques, Hôpital Général de Loandjili, B.P. 8122, Pointe-Noire, Congo; Institut Supérieur des Sciences de la santé, Université Hassan 1er Settat, B.P. 577, Settat, Maroc

**Keywords:** Specific-genotype, HPV, HSIL, ICC, Southwestern Congo

## Abstract

**Background:**

Knowledge on HPV prevalence and genotype distribution in HSIL and ICC is highly essential for the introduction of an effective vaccination program and appropriate epidemiological monitoring of viral ecology before and after vaccination in Congo. This study aimed to determine the specific-HPV genotypes in HSIL and ICC among women in southwestern Congo.

**Methods:**

125 archival paraffin-embedded biopsy collected between 2008 and 2012 and histologically diagnosed were investigated. DNA extraction was performed using the phenol/chloroform method. HPV search was performed by nested-PCR using MY09/MY11 and GP5+/GP6+ consensus primers followed by direct sequencing.

**Results:**

The mean age of participants was 44.3 ± 8.2 years. Overall, HPV prevalence was 89.6% (112/125) with all high-risk genotypes. HPV-DNA was detected in 81.5% (53/65) of HSIL and 98.3% (59/60) of ICC. HPV 16 the most common genotype was detected in 47.1% (25/53) of HSIL and 52.5% (31/59) of ICC. Other types identified were: HPV 33 (22.6%), HPV 18 (15%), HPV 31 (11.3%) and HPV 69 (3.7%) in HSIL, and HPV 33 (28.8%), HPV 18 (11.8%), HPV 31 (5%) and HPV 35 (1.7%) in ICC. Knowing that the ADC accounted for 6.7% (4/60) of ICC cases, HPV 18 was identified in 25% (1/4) of these cases against 75% (3/4) for HPV 16.

**Conclusion:**

Our study showed that HPV 16, 33, 18 and 31 were the four most common genotypes in women with HSIL and ICC. These findings indicate that current vaccines against HPV could help to reduce the burden of cervical cancer in Congo.

## Background

Cervical cancer (CC) is a major public health problem worldwide. Invasive cervical cancer (ICC) is the third most common carcinoma among women in the world, representing 8.8% of all cancers. Worldwide 560,505 new cases of CC are predicted to occur by 2015. More than 459,616 women aged less than 65 years will be affected. The number of deaths is estimated at 284 902 women a year with 191,833 women aged less than 65 years [1].

In sub-Saharan Africa (SSA), 34.8 (around 75 141) new cases of cervical cancer are diagnosed per 100 000 women annually, and 22.5 (around 50 233) per 100 000 women die from the disease [1, 2]. Rates of cervical cancer vary considerably in different sub-regions and are ranked first or second (after breast cancer) in all individual SSA countries. Guinea, Zambia, Tanzania, Malawi, and Mozambique have some of the highest ICC incidence rates in the world at >50 per100 000 women [2, 3].

The Republic of Congo holds also some of the highest incidence and mortality rates of CC in SSA. The Age-standardized incidence rate is 25.2 per 100 000 women (14.2 in the world and 30.6 in Central Africa). The Age-standardized mortality rate is 13.0 per 100 000 women each year in the Congo (6.8 in the world and 22.2 in Central Africa) [4].

Human papillomavirus (HPV) infection has been identified as the primary cause of CC and the third most common cause of cancer-related death among women. HPV-DNA has been found in approximately 100% of CC cases [5]. Currently more than 150 HPV are well characterized. Thereby, about 40 have a high tropism of anogenital tract. The International Agency for Research on Cancer [6] has defined twelve genotypes as high-risk (HR) oncogenic (HPV 16, 18, 31, 33, 35, 39, 45, 51, 52, 56, 58, and 59), One probable oncogenic risk assigned to genotype 68, and possible oncogenic risk assigned to the types: HPV 26, 53, 66, 67, 70, 73, 82, 30, 34, 69, 85, and 97. Low-risk (LR) genotypes that cause benign lesions and warts are (HPV 6, 11, 28, 32, 40, 42, 43, 44, 54, 55, 57, 61, 62, 71, 72, 74, 81, 83, 84, 86, 87, and 89) [1, 7, 8].

Although, most HPV infections are transient and disappear within 2 years without using any treatment [9], high-risk HPV types can cause persistent infection and are significantly associated with high-grade cervical lesions and cancer [10].

Moreover, two HPV vaccines have been developed, a bivalent for HPV-16 and HPV-18 and a quadrivalent for HPV-16, HPV-18, HPV-6 and HPV-11, capable of protecting in an effective way against an infection caused by these types [11, 12]. Although, these vaccines also confer significant immunity against other HPV types, the efficiency of cross-protection is less than 100% [13, 14].

Although the distribution of high-risk types in High-grade Squamous lesions (HSIL) and ICC shows little variation among regions of the world, [15–17], perfect knowledge of these types is essential for the introduction of an effective vaccination program and for better monitoring of viral ecology before and after vaccination in a less studied population. This information is also needed to assess the vaccine benefit on cervical cancer prevention in the population.

Until now, no study has been conducted on this target population in the Republic of Congo. Thus, the aim of this study was to determine the prevalence and the HPV type’s distribution in HSIL and ICC among Congolese women in the southwestern part of the country.

## Methods

### Sample collection

One hundred and thirty six formalin-fixed paraffin-embedded (FFPE) biopsy samples collected between 2008 and 2012 where HSIL and ICC diagnosis has been indicated were anonymously selected from tumor Registry files of Histopathological Unit of General Hospital of Loandjili (GHL) in Pointe-Noire. Altogether only 125 (91.9%) archival FFPE samples were found, 65 HSIL and 60 ICC (including 56 invasive squamous cell carcinomas (SCC) and 4 adenocarcinoma (ADC)) from women aged 28-74 years (mean age: 44.3 ± 8.2 years). The mention of diagnosis and especially the availability of biopsy to the laboratory were the two major inclusion criteria. For the purposes of scientific research, the study was approved by the local ethics committee in the Health Sciences (Comité d’éthique de la Recherche en Sciences de la Santé, CERSSA) with an exemption from requiring consent from patients (study was carried out on archival material to be destroyed after a certain preservation time).

### DNA extraction

Three to five sections of FFPE tissue were cut on a microtome and deparaffinized with xylenes and washed with 70% ethanol at ambient temperature. DNA extraction was performed using the manual technique of phenol/chloroform used in the laboratory of Virology, Microbiology and Quality/Eco-toxicology and Biodiversity (LVMQ/ETB) of the Faculty of Sciences and Techniques, University Hassan II Mohammedia-Casablanca in Morocco after enzymatic treatment with proteinase K. The DNA was precipitated with 2/5 volumes of 7.4M ammonium acetate and 2 volumes of 100% ethanol. DNA pellet was subsequently washed with 70% ethanol, air-dried and then suspended again in 30 or 50mL of Ultra-pure PCR water nuclease-free (Bioline, UK), thereafter stored at 20°C until further use.

### HPV-DNA detection and genotyping

After extraction, DNA concentration was evaluated in NanoDrop 8000 Spectrophotometer (Nanodrop Technologies, Wilmington, DE, USA). A 268-base-pair fragment of the housekeeping β-globin gene was amplified using the GH20/PCO4 primer set to evaluate quality and integrity of DNA extract as previously described [18]. All amplifications were carried out with 100ng/μL of DNA concentration in a Perkin Elmer 2400 GeneAmp PCR thermal Cycler (Scientific Support, Inc, Hayward, CA). DNA from the SiHa cell line was used as positive PCR control and Ultra-pure PCR water nuclease-free (Bioline, UK) as negative PCR control.

The samples were analyzed for HPV-DNA detection by nested-PCR using two consensus primer sets, i.e., MY09/MY11 and GP5/GP6 as previously described [19]. To avoid contamination leading to false positive results, both PCR round were performed in separate room. All primer sequences are given in Table 1. Amplified PCR product was analyzed on 2% agarose gel, stained with ethidium bromide and visualized under UV light. The PCR products were identified on the basis of their predicted fragment size. HPV typing was performed at the Molecular Biology and Functional Genomics platform of Support Unit Scientific Research and Technology, National Centre for Scientific and Technical Research of Rabat (UATRS-CNRST, Rabat, Morocco). Direct sequencing was undertaken from the nested PCR product according to the BigDye Terminator v3.1 Cycle Sequencing Kit (PE/Applied Biosystems, Foster City, CA, USA) using the GP6+ antisense as primer. Purification of PCR products were carried out with the ExoSAP-IT^®^ clean up system (USB Corporation, Cleveland, USA) whereas, the sequencing products was done with a Sephadex column 50G (Pharmacia Biotech Co., Ltd, Uppsala Sweden). Electrophoresis migration was performed in a 16 capillary automated sequencer 3131XL/Genetic HITACHI Analyzer (PE/Applied Biosystems, Foster City, CA, USA) at a constant voltage of 12 KV.

**Table 1:** **Primers used for HPV detection and typing in this study**

Primers	Sequences (5’ to 3’)	Target gene	Amplicons length	Ref
GH2O	GAA GAG CCA AGG ACA GGT AC	b-Globin	268pb	Resnick et al., 1990 [18]
PCO4	CAA CTT CAT CCA CGT TCA CC			
MYI 1	GCM CAG GGW CAT AAY AAT GG	L1	450pb	Lee et al. 2009 [19]
MYO9	CGT CCM ARR GGA WAC TGA TC			
GP5+	TTT GTT ACT GTG GTA GAT ACT AC	L1	142pb	
GP6+	GAA AAA TAA ACT GTA AAT CAT ATT C			
	M = A + C, R = A + G, W = A + T, Y = C + T			

**Figure 1:**
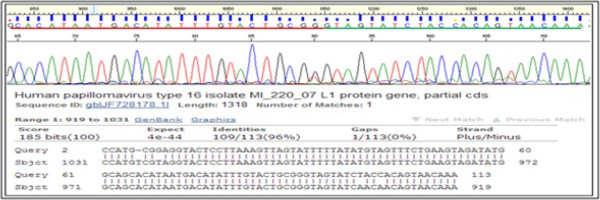
**Sequencing data of HPV genotypes: An example of electrophoregram sequence from HPV type 16 and its Basic Local Alignment Search (BLAST) analysis result.** Electrophoregram pointing the hyper variable region (underlined in red) of HPV L1 gene, downstream of the binding site of GP5+ primer (underlined in green, reading direction: from right to left).

The DNA sequences were aligned by Molecular Evolutionary Genetics Analysis (MEGA) software Version 4.0 (http://www.megasoftware.net) and results were analyzed using the BLAST algorithm (http://www.ncbi.nih.gov/BLAST). For each typing, we searched the hypervariable region from 34 to 50 bp downstream of the GP5+ binding site (Figure 1) able to accurately identify any HPV genotypes as described previously [19]. An identity of 90% or more with the sequences of the GenBank database (NCBI, national Institute of Health, Bethesda, MD, USA) was retained to confirm every type of HPV obtained. The Nucleotide sequences data set supporting the results of this article was included within the article as additional file published through the LabArchives database (DIO “10.6070/H4PZ56TF”).

## Results

### Overall HPV DNA prevalence

Detection of HPV DNA was performed in tumor samples of 125 Congolese women with HSIL or ICC histologically diagnosed. Overall, DNA was successfully amplified in all of the 125 (100%) samples tested (β-globin PCR). Our results showed that 112/125 (89.6%) of the total samples were positive for HPV-DNA. HPV-DNA was detected in 81.5% (53/65) of HSIL and 98.3% (59/60) of ICC (98.2% of SCC and 100% of ADC respectively).

### HPV type-specific prevalence

Six different genotypes have been identified, all were oncogenic high-risk types. HPV 16 the most common was detected in 47.1% (25/53, 95%IC: 34.4-60.3) of HSIL and 52.5% (31/59, 95%IC: 40.0-64.7) of ICC. Other types identified were in decreasing frequency: HPV 33 (22.6%, 95%IC: 13.4-35.5), HPV 18 (15%, 95%IC: 7.8-27.0), HPV 31 (11.3%) and HPV 69 (3.7%) in HSIL; HPV 33 (28.8%, 95%IC: 18.8-41.4), HPV 18 (11.8%, 95%IC: 5.8-22.5), HPV 31 (5%) and HPV 35 (1.7%) in ICC.

**Figure 2:**
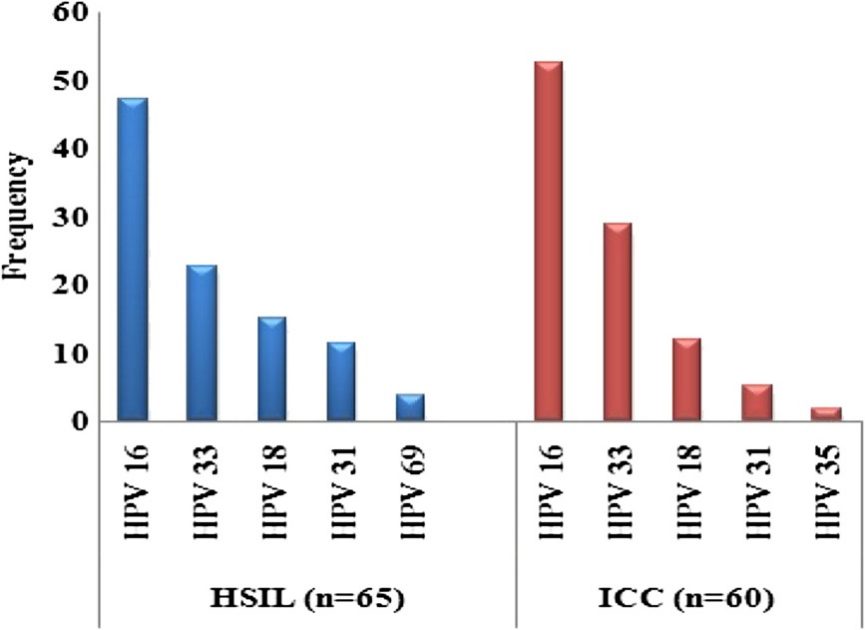
**HPV genotypes frequencies in HSIL and ICC among women from southwestern Congo.**

Knowing that the ADC accounted for 6.7% (4/59) of positive ICC cases, only 1 of out 4 (1.7%) of them was identified as HPV 18 against 3 of out 4 (5%) for HPV 16. All the results are reported in Table 2 and Figure 2.

**Table 2:** **Type-specific HPV and their distribution in paraffin-embedded biopsy specimens of HSIL and ICC cases from Congolese women, 2008–201**

Histological types	All HPV+	HPV genotypes n (%)									
	n(%)	16	95% IC	33	95% IC	18	95% IC	31	95.1C	69	35
HSIL (n = 65)	53(81.5)	25(47.1)	34.4-60.3	12(22.6)	13.4-35.5	8(15.0)	7.8-27.0	6(113)	5.3-22.6	2(3.7)	0
ICC (n = 60)	59(98.3)	31 (52.5)	40.0-64.7	17(28.8)	18.8.41.4	7(11.8)	5.8-22.5	3(5.0)	1.7-13.9	0	1(1.7)
SCC (n = 56)	55(98.2)	28 (50.9)	38.0-63.6	17(30.9)	20.3-44.0	6(10.7)	5.0-21.8	3(5.3)	1.8-14.8	0	1(1.8)
ADC (n = 4)	4(100)	3(75.0)	30.0-95.4	0		1(25.0)	4.5-69.9	0		0	0

95% CI = confidence interval for the four most common genotypes identified.

The five most common individual oncogenic HPV types found in ICC in our study were compared with profiles found in the World, African regions; Europe and North America according to the WHO/ICO HPV information center (Table 3).

**Table 3:** **The five most common HPV types among Congolese women with invasive cervical cancer in Southwestern Congo compared to the World, African regions, Europa and North America**

HPV position	Congo ^a^	World ^b^	Africa ^b^			Europa ^b^	North America ^b^
			North Africa	South Africa	Western Africa		
1^st^	HPV16 (52.5%)	HPV16 (56.7%)	HPV16 (58.5%)	HPV16 (52.1%)	HPV16 (40.5%)	HPV16 (58.8%)	HPV16 (54.6%)
2^nd^	HPV33 (28.8%)	HPV18 (15.9%)	NPV18 (19.9%)	HPV18 (10.7%)	HPV45 (13.7%)	HPV18 (16.3%)	HPV18 (17.1%)
3^nd^	HPV18 (11.8%)	HPV33 (4.6%)	HPV45 (8.0%)	HPV33 (9.1%)	HPV18 (10.9%)	HPV33 (4.4%)	HPV31 (5.3%)
4^th^	HPV31 (5.0%)	HPV45 (4.5%)	HPV31 (2.5%)	HPV31 (4.3%)	HPV58 (3.9%)	HPV45 (4.1%)	HPV45 (4.7%)
5^th^	HPV35 (1.7%)	HPV58 (4.4%)	HPV33 (2.4%)	HPV45 (3.3%)	HPV33 (3.2%)	HPV31 (4.1%)	HPV33 (3.6%)

(a) Data for Congo: present study.

(b) Source of information: WHO/ICO Information Centre on Human Papillomavirus (HPV) and Cervical Cancer [39].

## Discussion

The study reported here is the first of its kind among Congolese women with high-grade lesions and invasive cervical cancer in the aim of understanding the HPV prevalence and genotypes distribution in this target population groups in the southwestern part of the Country. Study was conducted retrospectively, using FFPE biopsies diagnosed as HSIL or ICC taken from 2008 to 2012, from GHL in Pointe-Noire. The GHL is the only hospital of south of the country to have a pathological laboratory. The Patients received in this laboratory come from different regions of the country and represents wide social and economic strata. Therefore, the results of our study could have particular interest in the area.

Worldwide, HPV prevalence in cervical cancer is reported to be 99.7% [5]. This prevalence is average 84.9% in HSIL and 87.2% in invasive cervical carcinoma [20]. In our study, 89.6% of the samples in general were HPV positive, of which 81.5% in HSIL and 98.3% in ICC. This findings corroborating with the literature data as reported in several countries especially in Africa where the prevalence is very high. Indeed, it was reported an average prevalence in Africa 85.1% and 93.9% in HSIL and ICC, respectively, with slight differences depending on the country [2, 21]. Some countries such as Senegal, South Africa and Kenya have a prevalence of 80.9%, 88.4% and 96.6% in HSIL respectively. Mozambique, Kenya, and Morocco for their part have a prevalence of HPV infection in the ICC of the order of 97.7%, 96.9% and 94.7% respectively [22, 23]. But also in Europe [24], as shown a recent study in Italy reported a 96.0% rate of HPV positivity in the ICC [25].

In our study, HPV 16 was the most common genotype found in HSIL (47.1%) and ICC (52.5%). These findings let appear a very high proportion of the infection by this genotype in our study population and are in keeping with other studies in the world [26–28]. These data show that infection with HPV 16 is a great risk of cancer development and particular attention should be devoted to these cases [29, 30].

We found also HPV 33 was the second most common types in HSIL (22.6%) and ICC (28.8%) followed by HPV18, 15% and 11.8%; HPV31, 11.3% and 5% in HSIL and ICC respectively. This profile although slightly different from those reported in sub-Saharan Africa [20, 31, 32], generally reflects the global distribution pattern of HPV in Africa in some meta-analysis studies [16, 33]. The type-specific distribution of HPV in799 cervical cancer biopsies from Africa showed that HPV 16 accounted for 50.2% of samples, HPV 18 for 14.1%, and HPV 45 for 7.9% [34]. The cumulative prevalence of HPV 16/18 in our study was 62.1% (47.1%/15%) and 64.3% (52.5%/11.7%) in HSIL and ICC respectively. Our results are lower than the global average which is 70% for both types [26, 27]. However, these results confirm the low prevalence of these two types combined in Africa compared to other regions of the world [16, 28].

In his study of women with HIV in Pointe-noire, Alidjinou using the Hybrid Capture II (HC2) method had found a prevalence of 70.9% of combined HPV16/18/45 [35]. Knowing that the probe (probe HC2 16/18/45) used by the latter allowed only to make a global typing, these proportions would be comparable to ours if he had used a specific genotyping method especially as we have not found any HPV 45 in our study. However ours findings while corroborating with those reported in the literature shows that current prophylactic vaccines against HPV would play a significant role in reducing the burden of cervical cancer in Congo.

In addition, although often diagnosed in the ADC, on 4 cases of our study, only a case was positive by HPV 18. This result indicates that the genotype 16 remains dominant whatever the histological type of the cancer. Frequently diagnosed among the first five genotypes in cervical cancer in the world, we found in our study a very high rate of HPV 33 in both HSIL (22.6%) than in ICC (28.8%), making it the second most prevalent type in our study population. This trend was also found with HPV 31 with frequencies of 11.3% and 5% in HSIL and ICC respectively. Admittedly lower degree, the high frequencies of these two types has been reported in several studies in Europe [17, 36]. In our study, these results could be explained by the fact that the study area is open to a significant population mixing related to the industrial and oil activities. In the literature, it is reported the high proportion of 4% to 6% [17, 37] of HPV35 in the ICC cases in SSA compared to another region in the world where the proportion is lower, ranging from 1% to 3% [16, 21]. In our study, 1 out of 60 cases was identified HPV-35 positive. Further studies with a larger sample are needed to assess the importance of this type in our population especially with the viral ecological change due to the vaccination against HPV 16/18. We also report the identification of HPV 69 (3.7%) in a relatively high proportion in HSIL but absent in the ICC. Although classified in the possible probable high risk types [1], its high proportion could reflect its oncogenic potential in cervix. An epidemiological surveillance would be necessary for this kind of genotype in our population after the introduction of vaccination against type 16 and 16.

In sum, the pattern of distribution of HPV in HSIL and ICC in southwestern Congo is as follows: HPV16, HPV33, HPV18, HPV31, HPV69 and HPV35. Note the notable absence of HPV45 which is among the five frequently identified worldwide genotypes. Our study showed for the first time a high prevalence of HPV in HSIL and ICC in women attending GHL in Congo. Early detection of infection for high-risk types could give physicians the possibility of more effective management of the disease [38]. This study has also shown the distribution of HPV genotype in HSIL and ICC, reflecting the local epidemiology of HPV transmission in southwestern Congo populations. Thus; the burden of cervical cancer in our country must be supported by the implementation of primary prevention through vaccination but also a real organized cytology screening program.

## Conclusion

The findings of this study are very significant because they provide, for the first time, baseline information on the HPV type’s distribution in HSIL and ICC in the Republic of Congo, which may guide the development of CC prevention and control programs in the country. These findings also indicate that HPV vaccination might be beneficial among Congolese population.
